# A new species of the tarantula genus *Haplocosmia* (Araneae, Theraphosidae) from Tibet, China

**DOI:** 10.3897/BDJ.10.e82682

**Published:** 2022-03-23

**Authors:** Ye-Jie Lin, Xunyou Yan, Shuqiang Li

**Affiliations:** 1 Hebei Key Laboratory of Animal Diversity, College of Life Science, Langfang, China Hebei Key Laboratory of Animal Diversity, College of Life Science Langfang China; 2 Institute of Zoology, Chinese Academy of sciences, Beijing, China Institute of Zoology, Chinese Academy of sciences Beijing China

**Keywords:** Asia, diagnosis, taxonomy, type

## Abstract

**Background:**

*Haplocosmia* Schmidt & von Wirth, 1996 is a small genus distributed in the Himalayas that includes two species. This genus has not been found in China (Li et al. 2021, Yao et al. 2021, Zhao et al. 2022).

**New information:**

A new species of the genus *Haplocosmia* is described from China: *Haplocosmiasherwoodae* **sp. n.** from Tibet. Photos and a morphological description of the new species are given. The type specimen of the new species is deposited in the Institute of Zoology, Chinese Academy of Sciences in Beijing (IZCAS).

## Introduction

The spider family Theraphosidae Thorell, 1869 includes 1031 species in 153 genera, with two species in the genus *Haplocosmia* Schmidt & von Wirth, 1996: *H.himalayana* (Pocock, 1899) from the Himalayas and *H.nepalensis* Schmidt & von Wirth, 1996 from Nepal (Li 2020, World Spider Catalog 2021).

*Haplocosmia* can be distinguished from all Selenocosmiine genera by the undivided spermathecae, thorn-like setae on the chelicerae prolaterally, scopula present on tarsi I–IV, scopula on tarsus IV divided by setae, a procurved fovea, procurved anterior eye row and butterknife-like stridulating setae on the prolateral surface of the maxillae (Schmidt and von Wirth, V. 1996).

Here, one new species of the genus *Haplocosmia* from China, *Haplocosmiasherwoodae* sp. n. from Xigaze, Tibet, is reported.

## Materials and methods

The specimen was preserved in 75% ethanol. Genitalia were cleared in trypsin enzyme solution to dissolve non-chitinous tissues. The specimen was examined under a LEICA M205C stereomicroscope. Photomicroscope images were taken with an Olympus C7070 zoom digital camera (7.1 megapixels), stacked with Helicon Focus 6.7.1 and processed in Adobe Photoshop CC 2018.

The terminology used in the text and figures follows Bertani (2000) and Sherwood et al. (2021). All measurements are in millimetres. Eye sizes were measured as the maximum diameter from either the dorsal or frontal view. Leg measurements are given as follows: total length (femur, patella, tibia, metatarsus, tarsus).

Abbreviations: **A**, apical keel; **ALE**, anterior lateral eyes; **AME**, anterior median eyes; **BL**, Basal lobe of retrolateral embolus keel; **MOA**, median ocular area; **PLE**, posterior lateral eyes; **PME**, posterior median eyes; **PS**, prolateral superior keel. The type material is deposited in the Institute of Zoology, Chinese Academy of Sciences in Beijing (**IZCAS**).

## Taxon treatments

### Haplocosmia sherwoodae

Lin & Li sp. n.

E4FCBBD8-D8B7-5DF1-B72E-CF56EEE66F33

8E5D1B9D-C956-4B10-B7EC-7A41C30208D0

#### Materials

- **Type status:** Holotype. **Occurrence:** individualID: IZCAS-Ar42679; **Taxon:** scientificName: *Haplocosmiasherwoodae*; order: Araneae; family: Theraphosidae; genus: Haplocosmia; **Location:** country: China; stateProvince: Tibet; county: Xigaze; municipality: Nyalam; locality: from Zham to Guomen; verbatimElevation: 2333; verbatimLatitude: 27.9785°N; verbatimLongitude: 85.9782°E; **Event:** year: 2021; month: 6; day: 13; **Record Level:** institutionCode: IZCAS

#### Description

**Figure 1.**
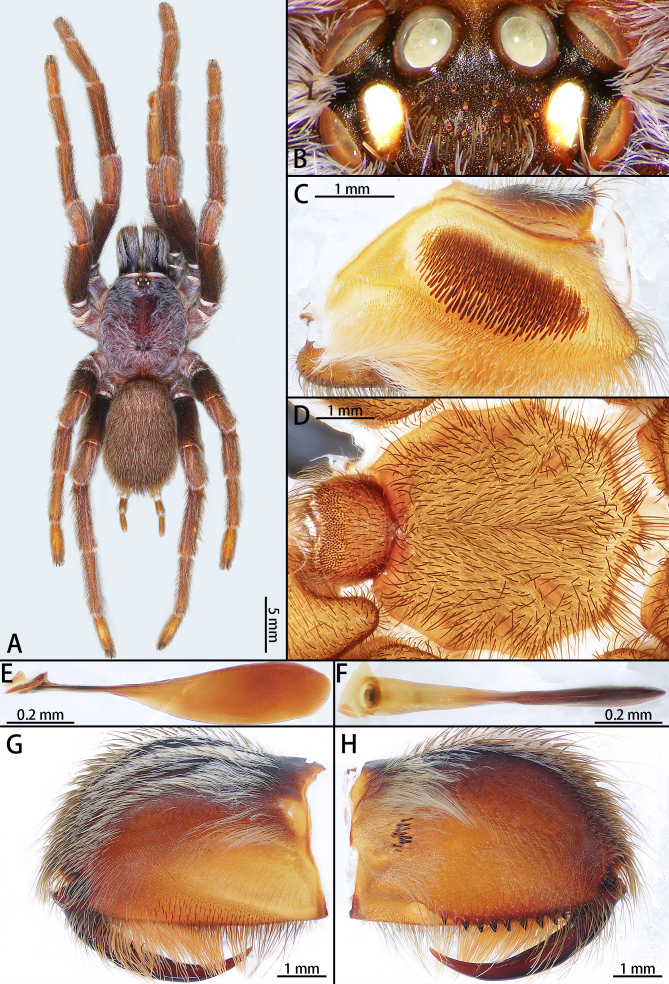
*Haplocosmiasherwoodae* sp. n., holotype male. **A** male habitus, dorsal; **B** ocular tubercle; **C** right palp maxillae; **D** sternum; **E** stridulatory lyra, lateral view; **F** same, ventral view; **G** chelicerae, retrolateral view; **H** same, prolateral view.

**Figure 2.**
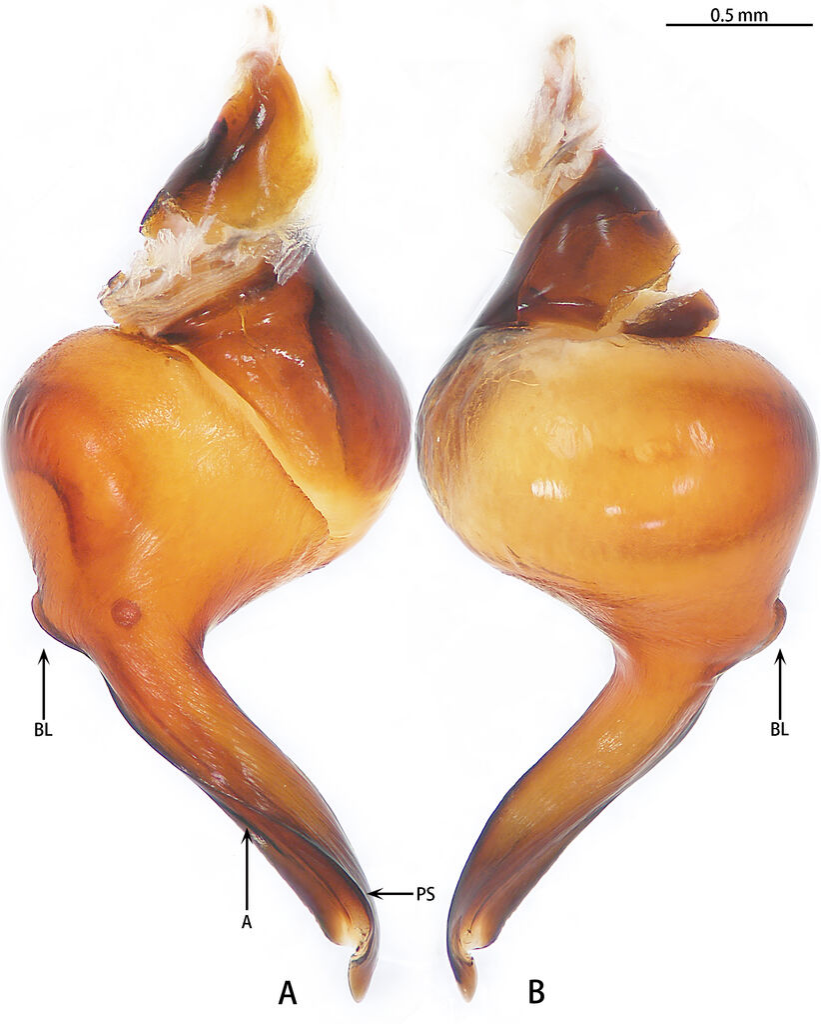
*Haplocosmiasherwoodae* sp. n., holotype, right palp bulb, rotated horizontally. **A** retrolateral view; **B** prolateral view.

**Figure 3.**
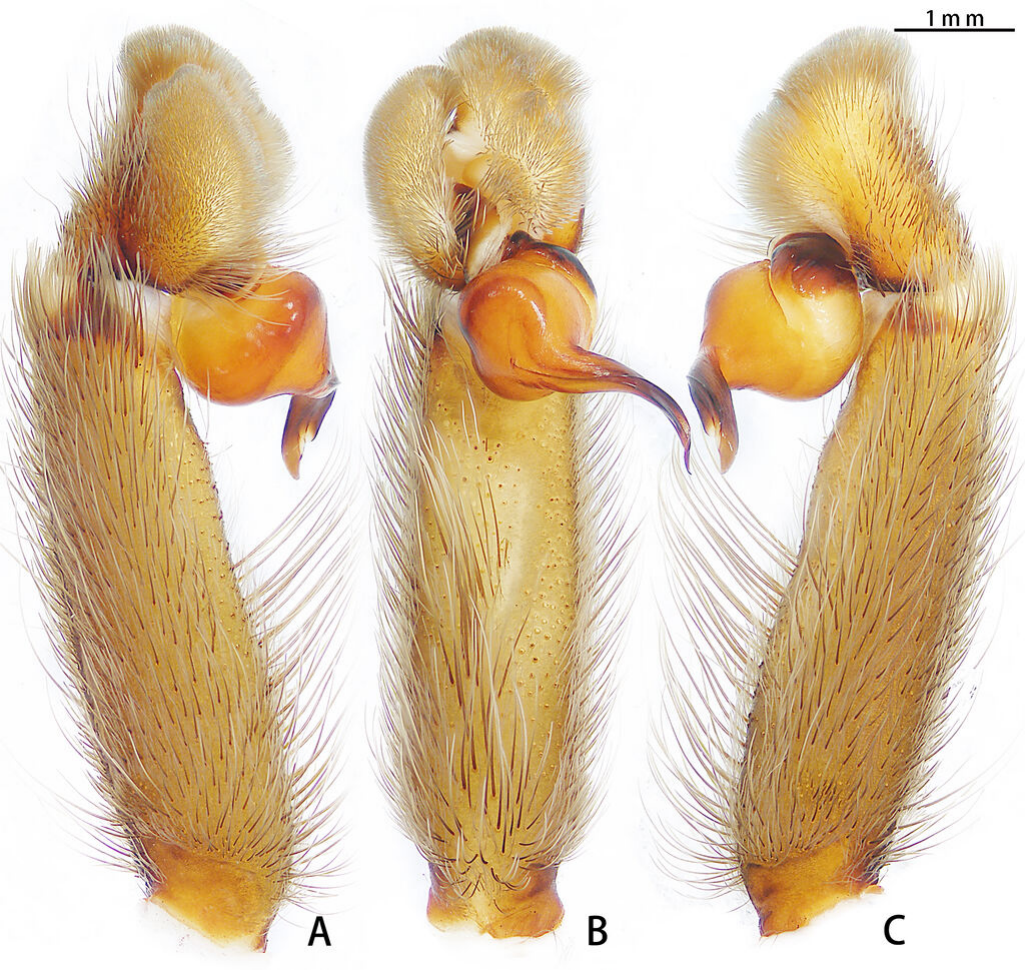
*Haplocosmiasherwoodae* sp. n., holotype, left palp. **A** prolateral view; **B** ventral view; **C** retrolateral view.

Male (holotype, IZCAS-Ar42679) (Fig. 1A–H, Fig. 2A and B, Fig. 3A–C). Carapace 8.09 long, 6.73 wide, brown with long white-purple setae. Opisthosoma brown, with long white and black hair (Fig. 1A). Eye group 1.28 long, 0.93 wide (Fig. 1B). MOA 0.71 long, anterior width 0.72, posterior width 0.96. Eye sizes and interdistances: ALE 0.40, AME 0.33, PLE 0.32, PME 0.37; ALE–AME 0.07, AME–AME 0.11, PLE–PME 0.04, PME–PME 0.56. Fovea slightly procurved. Chelicerae dark brown, with white hair, eighteen intercheliceral pegs, eight of them obvious, with prolateral keeled ridge. promargin with 10 stout teeth, basomesally with 24 denticles (Fig. 1G, H). Labium wider than long, with 286 cuspules. Sternum yellow brown with three pairs of sigilla (Fig. 1D). Legs with long and short setae. Tarsus I–IV with scopula and many setae laterally swollen, scopula on tarsus IV divided by setae, all tarsi 2 claws, without denticle. Leg measurements: I 20.43 (5.61 + 2.44 + 5.07 +3.68 + 3.63), II 22.27 (5.84 + 2.94 + 5.92 + 3.73 + 3.84), III 17.09 (4.51 + 2.07 + 3.63 + 3.82 + 3.06), IV 23.69 (6.30 + 2.67 + 5.56 + 5.83 + 3.33). Leg formula: 4213.

Male palpal bulb (Fig. 2A and B, male palp with bulb Fig. 3A–C). Bulb oval, embolus bow-shaped, slightly curved 180°, with A, PI and PS. Distal edge of embolus relatively flat. Maxillae with lyra setae ventrally (Fig. 1C). Stridulatory lyra paddle-shaped (Fig. 1E and F).

Female unknown.

#### Diagnosis

*Haplocosmiasherwoodae* sp. n. is found at 2356 m elev., in a higher, more montane environment, close to areas of permanent snowfall. (vs. *H.nepalensis* Schmidt & von Wirth, 1996 which is only known to occur at an elevation of < 1200 m in Kathmandu (Schmidt and von Wirth, V. 1996, West et al. 2012). The male of *H.sherwoodae* sp. n. is similar to *H.nepalensis* by the shape of the bulb, but it can be distinguished from *H.nepalensis* by the PS keel elongate, with distinct BL, originating ventrally at ventro-medial aspect of palpal bulb (vs. PS non-elongate, without BL and not originating ventrally at ventro-medial aspect of palpal bulb in *H.nepalensis*) and the length of embolus base to the width of the bulb being 3:1 (vs. 2:1 in *H.nepalensis*) (Figs. 2A and B).

#### Etymology

The species is named after Miss Danni Sherwood, who confirmed the validity of the new species; noun (name) in genitive case.

#### Distribution

Known only from the type locality.

## Supplementary Material

XML Treatment for Haplocosmia sherwoodae
